# Early-Life β-Lactam Exposure and the Developing Microbiome: Clinical Relevance and Controversies

**DOI:** 10.3390/microorganisms14020440

**Published:** 2026-02-12

**Authors:** Nilima Rajpal Kundnani, Abhinav Sharma, Mihaela Codrina Levai, Lucretia Marin-Bancila, Doina Georgescu, Loredana Botas, Sorin Dan Chiriac, Mihaela Valcovici, Mihaela-Diana Popa

**Affiliations:** 1 University Clinic of Internal Medicine and Ambulatory Care, Prevention and Cardiovascular Recovery, Department VI-Cardiology, “Victor Babes” University of Medicine and Pharmacy, 300041 Timisoara, Romania; 2 Research Centre of Timisoara, Institute of Cardiovascular Diseases, “Victor Babes” University of Medicine and Pharmacy, 300041 Timisoara, Romania; 3 Doctoral School, “Victor Babes” University of Medicine and Pharmacy, 300041 Timisoara, Romania; 4 Discipline of Medical Communications, Department 2-Microscopic Morphology, “Victor Babes” University of Medicine and Pharmacy, 300041 Timisoara, Romania; 5 Research Center for Medical Communication, “Victor Babes” University of Medicine and Pharmacy, Eftimie Murgu Square No. 2, 300041 Timisoara, Romania; 6 “Victor Babes” University of Medicine and Pharmacy, Eftimie Murgu Square No. 2, 300041 Timisoara, Romania; 7 Department V, Internal Medicine I—Discipline of Medical Semiology I, Center of Advanced Research in Cardiology and Hemostasology, “Victor Babes” University of Medicine and Pharmacy, 300041 Timisoara, Romania; 8 Discipline of Surgery III, Department X, Faculty of Medicine, “Victor Babes” University of Medicine and Pharmacy, 300041 Timisoara, Romania; 9 Department VI-Cardiology, “Victor Babes” University of Medicine and Pharmacy, 300041 Timisoara, Romania; 10 Department of Microbiology, “Victor Babes” University of Medicine and Pharmacy, 300041 Timisoara, Romania

**Keywords:** β-lactam, dysbiosis, antibiotics, microbiome, perinatal exposure, skin sources of transmission

## Abstract

Antibiotic-induced dysbiosis has been increasingly implicated in a range of pediatric outcomes, yet the concept remains variably defined and often inconsistently applied. The purpose of this review is to provide an overview and critical evaluation of the available data regarding the effects of early-life exposure to β-lactam antibiotics on the developing microbiome. We conducted a narrative review of experimental and epidemiological studies examining β-lactam exposure during pregnancy, the perinatal period, and early childhood was conducted. β-lactams induce reproducible alterations in microbial composition, diversity, and metabolic function, including decreases in *Bifidobacterium* and *Lactobacillus* and a relative increase in *Enterobacteriaceae* and other facultative anaerobes, especially in early life. Reduced microbial diversity and changed short-chain fatty acid-producing taxa often accompany these compositional changes. However, associations with immune, metabolic, and neurodevelopmental outcomes are heterogeneous and frequently confounded by indication host-related factors. Evidence for causality in humans remains limited despite strong mechanistic support from animal models. Current data support cautious interpretation, even though β-lactam-associated microbiome perturbations may contribute to disease susceptibility during vulnerable developmental windows. While mechanistic and longitudinal evidence continues to develop, antibiotic stewardship focused on appropriate indication and duration is still crucial.

## 1. Introduction

The human microbiome plays a crucial role in maintaining immune, metabolic, and neurodevelopmental balance. Changes in microbial composition and function, often referred to as dysbiosis, have been increasingly associated with various pediatric conditions [1].

Numerous factors, such as birth weight, gestational age, feeding habits, and mode of delivery, affect the development of the early-life microbiota [2]. Low birth weight and preterm birth are linked to reduced diversity, delayed microbial maturation, and increased susceptibility to dysbiosis, which may intensify the effects of environmental exposures [3]. Also, antibiotics, particularly β-lactams, are among the most commonly prescribed medications in early life and during pregnancy. However, their impact on the developing gut microbiota raises concerns about both short-term and long-term health effects. It is essential to understand how exposure to β-lactams disrupts microbial communities to balance clinical benefits with potential risks [4,5].

This review aims to summarize current evidence regarding β-lactam–induced dysbiosis in children and infants, focusing on its mechanisms, clinical outcomes, and implications for pediatric health.

## 2. Methods

This article is a narrative review that synthesizes current evidence on the effects of β-lactam antibiotics on the pediatric microbiome, including mechanistic insights, clinical outcomes, and short- and long-term consequences. A targeted literature search was performed in ScienceDirect, Scopus, PubMed and Google Scholar using relevant keywords (e.g., “beta-lactam,” “antibiotics,” “dysbiosis,” “microbiome,” “infants,” “perinatal exposure”), and articles were selected based on relevance, novelty, and methodological quality. We included animal studies, peer-reviewed human studies, cohort studies, and relevant review articles that link microbiome changes to antibiotic exposure. We excluded those written in languages other than English.

This review emphasizes both well-established findings and areas of controversy or uncertainty, aiming to provide a comprehensive overview and identify gaps for future research.

### 2.1. Shaping the Pediatric Gut Microbiota

**Figure 1:**
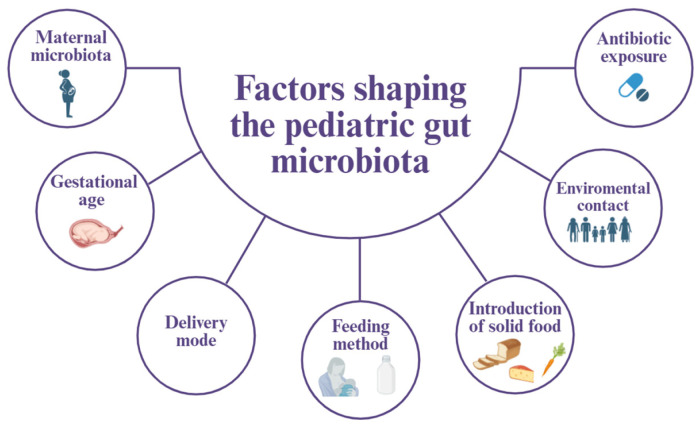
Key factors shaping the development of the pediatric gut microbiota. The pediatric gut microbiota is shaped by a complex interplay of prenatal, perinatal, and postnatal factors. Maternal microbiota and gestational age influence initial microbial seeding, while mode of delivery and feeding method further modulate early colonization patterns. Environmental contact and the introduction of solid foods contribute to microbial diversification during infancy. Antibiotic exposure, particularly during critical developmental windows, can disrupt microbial composition and delay microbiota maturation, with potential short- and long-term consequences for host immune and metabolic development.

The gut microbiota plays essential roles in human physiology and undergoes dynamic changes across the lifespan. Its major phase of assembly begins at birth, when initial colonization is shaped by maternal microbial exposures and the early-life environment. During infancy, compositional and functional maturation occurs rapidly, supported by successive waves of microbial taxa that progressively transform the neonatal ecosystem into a more complex, adult-like community. A broad range of prenatal and postnatal factors—including maternal microbiota, gestational age, mode of delivery, feeding practices, early antibiotic exposure, and environmental contacts—influence both the direction and pace of microbial development in this critical window (Figure 1) [2,3,6].

A few early studies reported bacterial DNA in samples such as meconium or amniotic fluid, which led to questions about whether the intrauterine environment is truly sterile [7]. However, these findings were methodologically limited and prone to contamination [8]. Subsequent high-quality studies have shown that most earlier signals were contaminants rather than evidence of in utero colonization [9], and current data do not support the presence of a fetal or placental microbiota in healthy pregnancies [10].

In early life, the maternal microbiome plays a central role in seeding the infant gut. The first microbial communities acquired by the newborn originate from maternal body sites such as the skin, oral cavity and vagina, supporting a clear pattern of vertical microbial transmission [11]. The newborn’s first major exposure to bacteria occurs at birth and is strongly influenced by the mode of delivery. Vaginally delivered infants acquire microbial communities that closely resemble their mother’s vaginal microbiota—typically enriched in *Lactobacillus*, *Prevotella*, and *Sneathia* species. In contrast, infants born by caesarean section are primarily colonized by skin-associated taxa such as *Staphylococcus*, *Corynebacterium* and *Propionibacterium*, reflecting maternal skin sources of transmission [12]. These delivery-related differences in early microbial colonization have been associated with an increased susceptibility to certain immune-mediated and metabolic conditions among infants born by caesarean section, compared with those delivered vaginally [13]. As site-specific microbial communities establish, the vertically transferred maternal vaginal microbiota may help occupy early ecological niches, theoretically limiting opportunities for colonisation by exogenous pathogens. This concept is particularly relevant given reports of community-associated MRSA infections even among otherwise healthy newborns [14]. Caesarean delivery has been associated with altered early gut colonisation patterns, including reduced early exposure to beneficial taxa such as *Lactobacillus* and *Bifidobacterium* [12]. Epidemiological data further indicate that infants born by caesarean section have a higher risk of developing asthma and allergic disease [15].

Breastfeeding is a key driver of early gut microbiota development. Human milk not only delivers beneficial bacteria but, more importantly, contains human milk oligosaccharides (HMOs) that selectively promote the growth of taxa such as *Bifidobacterium*. In addition, the bioactive components of breast milk—including immunoglobulins and antimicrobial factors—help limit the expansion of potentially harmful bacteria [16]. Human milk contains multiple immunomodulatory factors—including erythropoietin, interleukin-10, epidermal growth factor and transforming growth factor-β1—that contribute to regulating intestinal inflammation and supporting mucosal immune maturation in early life [17]. Compared with formula-fed infants, breastfed infants typically harbor higher abundances of *Bifidobacterium* and *Lactobacillus*. This microbial profile promotes greater production of short-chain fatty acids (SCFAs) and a lower intestinal pH, creating conditions that can inhibit the growth of several pathogenic organisms [1]. Formula-fed neonates tend to develop a more diverse gut microbiota, characterized by higher abundances of facultative anaerobes and a broader range of taxa—including *Escherichia coli*, *Clostridium difficile*, *Bacteroides*, and *Prevotella*—compared with the more *Bifidobacterium*-dominated profiles seen in breastfed infants Notably, even partial supplementation with formula can shift the gut microbiota of breastfed infants toward patterns typically observed in formula-fed neonates [18]. Although breastfed infants display lower bacterial diversity, their gut microbiota is enriched in genes required for the breakdown of HMOs. Metagenomic analyses further indicate that these microbial functions engage more extensively with host immune-, metabolic- and biosynthetic-related pathways compared with the microbiota of formula-fed infants [13,19]. Gut microbial diversity increases markedly after the introduction of solid foods, and by around three to five years of age the overall community structure resembles that of adults [18].

The newborn’s immediate environment also contributes microbial exposures that can seed different body sites. Early-life bacterial exchange can occur through contact with shared surfaces, household objects and the indoor air microbiome, reflecting the continuous microbe flow between humans and their surroundings [20]. In addition, greater gut microbial diversity has been associated with exposure to older siblings, likely reflecting increased microbial exchange within the household environment [21]. Studies have shown that individuals living in the same household tend to share similar gut, skin and oral microbiota profiles, reflecting extensive microbial exchange within families [22]. Early-life exposure to household pets has been associated with immunomodulatory effects, and experimental data show that microbial inputs from pet-containing environments can promote gut *Lactobacillus* enrichment and enhance airway immune defenses against allergens and viral infections [23].

Antibiotics are among the most frequently prescribed medications in childhood, making it essential to understand their impact on the developing gut microbiota [4]. Research shows that antibiotic exposure in infancy disrupts the stability of the gut microbiota, reduces microbial diversity, and promotes the expansion of bacteria carrying antibiotic-resistance genes [24]. Antibiotic exposure in the first days of life can delay the colonisation of the gut by *Bifidobacterium*. The resulting reduction in *Bifidobacterium* and *Bacteroides* species is often accompanied by an overrepresentation of enterobacteria and enterococci, a pattern associated with lower microbial diversity and with less favourable immunological and metabolic outcomes later in infancy [3,25]. To facilitate visualization of the reported microbiome alterations across early-life conditions and β-lactam exposure, the main bacterial taxa described in the literature are summarized in Table 1.

### 2.2. Dysbiosis Induced by β-Lactams

Antibiotics are one of the most prescribed medications and have numerous benefits [31]. Out of them, β-lactam antibiotics rank among the most commonly recommended antimicrobial medicines for children, owing to their extensive efficacy and advantageous safety profile [32]. The β-lactam antibiotics is a family of bactericidal drugs structurally related containing the β-lactam ring in their chemical structure. They are classified in penicillins, cephalosporins, carbapenems, penems (also known as thiopenems) and monobactams [33].

Their antimicrobial spectrum varies across subclasses but generally encompasses a wide range of Gram-positive and Gram-negative bacteria [34]. Narrow-spectrum penicillins, such as benzylpenicillin and phenoxymethylpenicillin, mainly target *Streptococcus* and *Staphylococcus* species, whereas aminopenicillins (amoxicillin, ampicillin) and many cephalosporins exhibit extended activity against Enterobacteriaceae, *Haemophilus influenzae*, and *Neisseria* species [35]. Broad-spectrum β-lactams, including third-generation cephalosporins and carbapenems, further expand coverage to anaerobes and multidrug-resistant Gram-negative organisms [36].

Despite its clinical benefits, this wide range of antibacterial activity is also the primary cause of microbiome disruption. The gut’s microbial diversity is greatly diminished by β-lactams, which impact both commensal and pathogenic bacteria [37]. Species like *Bifidobacterium* and *Lactobacillus*, which are significant producers of SCFAs, are particularly vulnerable [38], in contrast to species like *Escherichia*, *Enterobacter*, *H. pylori*, and *C. difficile*, which often proliferate due to less competition [27,39]. The extent of dysbiosis depends on the spectrum, dosage, duration, and route of administration [5,40,41]: for example, oral amoxicillin–clavulanate has been shown to cause profound, sometimes prolonged, shifts in microbial composition compared to narrow-spectrum agents [42].

Although all β-lactam antibiotics share the same core mechanism—blocking bacterial cell wall synthesis through inhibition of penicillin-binding proteins—the extent and pattern of microbiome disruption vary substantially across subclasses. Based on the research included in this narrative review, Table 2 lists the primary β-lactam subclasses, their antimicrobial targets, and their documented impacts on the gut microbiome.

### 2.3. Clinical Evidence in Antibiotic Use in Children

Across pediatric studies, β-lactam exposure is associated with a reduction in overall microbial diversity and a selective depletion of beneficial commensals such as *Bifidobacterium* and *Lactobacillus*. These changes suggest ecological imbalance within the intestinal ecosystem.

#### 2.3.1. Age-Dependent Effects

β-lactam antibiotics are among the most often administered antibiotics during pregnancy [49]. Nevertheless, the usage of antibiotic therapy during pregnancy, lactation, and even infancy varies depending on the underlying illness, nation, and medical recommendations.

##### Pregnancy

Antibiotic exposure during pregnancy can affect fetal neurodevelopment, metabolism, and immunity through microbiome-mediated pathways. Prenatal antibiotic exposure has been associated with an increased risk of childhood asthma and atopic disease [50], although a large Danish registry suggested that part of this association may reflect inherited susceptibility to infections [51,52]. Maternal gut microbiota shapes fetal adaptive and innate immunity [53]. Asthma at age five has also been linked to maternal staphylococcal colonization and antibiotic use [52]. Maternal carriage of *Prevotella copri* seems to protect against food allergies, although aminoglycoside exposure may counteract this effect [54]. There are conflicting findings on other outcomes where intrapartum antibiotics do not appear to be a contributing factor, such as atopic dermatitis and caesarean birth [55,56].

Short-chain fatty acids (SCFAs), which come from the mother’s microbiota, are important for the embryo’s energy balance, fetal growth, and the development of its metabolism [57]. The evidence relating prenatal antibiotic exposure to juvenile metabolic outcomes is mixed: some cohorts suggest increased overweight risk at age 7 [58,59], while others show no overall connection with obesity [60,61,62]. Early-life obesity findings are also inconsistent [58,60,63], possibly due to heterogeneity in population, sample size, antibiotic regimen, and confounders [59]. Prenatal antibiotics have also been linked to reduced birth weight [57,64], which is a known risk factor for later obesity, poor glucose regulation, and diabetes, potentially due to rapid postnatal “catch-up growth” [65]. The altered microbiome makeup in diabetic children supports a mechanistic connection [66].

Through the microbiome–gut–brain axis, prenatal microbial alterations may also affect brain development [67]. Immunologic, endocrine, and neurological pathways are influenced by microbial signals [67,68,69]. Infancy is a crucial period for concurrent brain and microbiome development [70]. Exposure to antibiotics can interfere with these processes in a number of ways, including decreased maternal gut function, altered fetal nutritional availability and gut permeability [71,72], neonatal microbial imbalances that impact neurotransmitter pathways [72], and increased Hypothalamic–Pituitary–Adrenal axis (HPA) reactivity [73,74]. Although certain cohort and case–control studies showed an elevated risk, systematic evaluations found no overall relationship between perinatal antibiotics and autistic spectrum disorders (ASD) [74]. One study indicated a greater risk of cerebral palsy after intrapartum antibiotic prophylaxis, and postnatal exposure may slightly increase the risk of ASD [75]. Although microbiome-mediated impacts on metabolism and neurodevelopment are consistently supported by animal studies, solid human data are still inconsistent.

##### Perinatal Period

The most frequent cause of exposure to antibiotics during the perinatal period is the use of intrapartum antimicrobial prophylaxis (IAP). IAP is commonly used during C-section delivery and in Group B *Streptococcus*-positive (GBS) women before vaginal delivery.

IAP was widely used after guidelines were adopted, and a large U.S. population-based study assessing universal antenatal screening for GBS showed a sharp increase in maternal screening (from 48% to 85%). The incidence of early-onset GBS disease remained 0.32 per 1000 live births despite these interventions, with the majority of cases occurring in term infants and more than 60% coming from mothers who had tested negative for GBS before delivery [76]. These findings highlight that although perinatal antibiotic administration can reduce severe neonatal infections, exposure during this critical developmental window is highly prevalent and often administered broadly rather than selectively.

The Central European Longitudinal Studies of Parents and Children: The Next Generation (CELSPAC: TNG) study states that the oral bacteriome is also impacted by IAP exposure during the first 48 h after birth. But later in the first week of life, the effects of IAP appear to lessen. IAP influences early neonatal oral and gut bacterial colonization, but this effect seems to be more pronounced in the first few days and in transitional stool than in meconium [26].

Regarding differences between microbial composition and diversity of newborns born to mothers who received IAP, a systematic review from a Chinese-based population found significant differences in microbial composition. It was discovered that perinatal antibiotic exposure induced microbiota dysbiosis in both the mother’s vagina and the neonatal gut, with a notable decrease in the abundance of *Lactobacillus* spp. Furthermore, full-term newborns who were not exposed to antibiotics showed no evidence of early-onset sepsis, whereas at least one infant diagnosed with early-onset sepsis was observed among full-term or preterm newborns who received antibiotic exposure before birth [77].

However, there is still little evidence to support the negative effects of IAP on children’s health in long-term follow-ups.

##### Infancy

The impact of antibiotic exposure on the gut microbiome appears to be strongly influenced by the child’s age at the time of treatment. The first months of life represent a critical window for microbial colonization, immune education, and metabolic development. During this period, the gut ecosystem is still establishing its core bacterial communities, dominated initially by *Bifidobacterium* and *Bacteroides* species. Antibiotic interference at this stage, particularly in premature infants, can alter both the composition and function of the microbiota [78].

There is evidence that early exposure to antibiotics, especially in infants younger than 90 days, was linked to an increased likelihood of developmental delays by preschool age in fine motor skills, cognition, and communication skills [79].

Regarding β-lactams, they significantly disrupt the initial colonization patterns of gut microbes in infants. Infants who have been exposed to these antibiotics show notable changes in their microbial composition. Specifically, there is an increase in the levels of bacteria such as *Klebsiella*, *Enterococcus*, *Streptococcus*, *Alistipes,* and *Aeromonas*. At the same time, there is a decrease in beneficial early colonizers like *Escherichia–Shigella*, *Clostridium sensu stricto 1*, *Bifidobacterium*, and *Parabacteroides*. The magnitude and direction of these alterations varied according to treatment duration, β-lactam type (cefotaxime versus ampicillin/sulbactam), and delivery mode, with cesarean-delivered infants showing the greatest dysbiosis [80].

Also, infants exposed early to β-lactams often display lower microbial diversity compared to antibiotic-naïve peers as well as alterations in the resitome, defined as the collection of all antibiotic resistance genes present within the gut microbiota, including both expressed and potentially transferable elements. Importantly, resistome variability was linked to adverse neonatal outcomes such as early-onset sepsis and bronchopulmonary dysplasia, underscoring that early-life antibiotic exposure not only perturbs microbiome development but may also influence susceptibility to disease in preterm infants [54]. Although late administration of antibiotics also impacts the microbiota, it tends to affect microbiota at a later developmental stage that may be less critical [81].

Collectively, these findings underscore that the timing of antibiotic exposure is as important as its duration or spectrum, emphasizing the need for cautious antibiotic stewardship during the first years of life.

### 2.4. Health Consequences of β-Lactams Induced Dysbiosis

**Figure 2:**
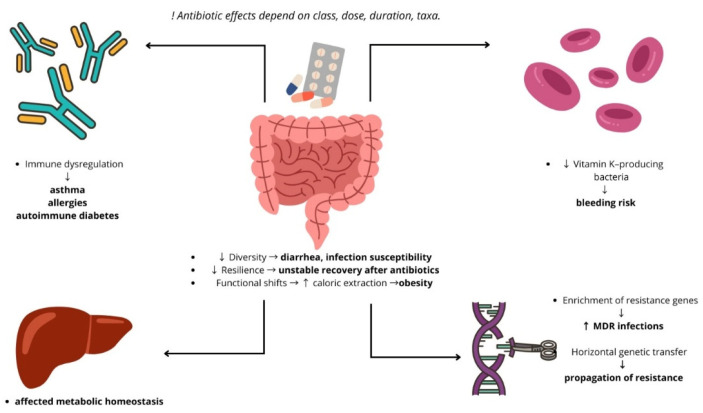
Antibiotic-induced disruptions in the gut microbiota and their systemic consequences. Schematic representation of how antibiotic exposure alters gut microbial composition and function, leading to downstream systemic effects. Antibiotic-induced dysbiosis is associated with immune dysregulation, altered metabolic homeostasis, enrichment of antimicrobial resistance genes, and impaired vitamin production, increasing the risk of bleeding and other adverse clinical outcomes. Adapted from Langdon et al. [87].

Antibiotics are consistently reported as one of the most commonly prescribed medication classes in children, accounting for a substantial proportion of outpatient pediatric prescriptions [4]. Although antibiotics are essential for treating bacterial infections, extensive evidence shows that early-life exposure can disrupt the assembly and maturation of the gut microbiota, which is particularly vulnerable during infancy [82]. This disruption can have profound consequences on host physiology and long-term health. In this section, we examine both the short-term and long-term effects of antibiotic-induced dysbiosis in children, including mechanistic insights and epidemiological associations (Figure 2).

Early childhood exposure to antibiotics is strongly associated with antibiotic-related dysbiosis, characterized by reduced microbial diversity and depletion of key commensal taxa. Such perturbations have been shown to alter immune development and to permit expansion of antibiotic-resistant bacteria [83,84,85,86]. Early-life alteration of host immunity and gut microbiota is associated with the emergence of immune-related and metabolic illnesses in later life [87]. When given to groups that are more likely to have gut microbiota dysbiosis, such as infants, obese infants, children with recurrent infections, and toddlers with allergic rhinitis, antibiotics alter the variety and composition of microbes, aggravating dysbiosis and having detrimental effects on health [88].

#### 2.4.1. Short-Term Effects

##### Antibiotic-Associated Diarrhea

Short-term disruption of the gut microbiota caused by β-lactam antibiotics is a well-recognized risk factor for antibiotic-associated diarrhea (AAD) in children. Aminopenicillins and cephalosporins frequently lead to acute diarrheal episodes, many of which involve infectious etiologies such as *C. difficile* or *Klebsiella oxytoca* [89,90,91]. Broad-spectrum β-lactam antibiotics—including aminopenicillins and oral cephalosporins—rapidly reduce gut microbial diversity and deplete obligate anaerobes such as *Bifidobacterium*. These shifts, detectable within days of exposure, weaken colonization resistance and create ecological niches that favor opportunistic pathogens [80,92,93,94]. Short-term AAD diarrhea is one of the most consistently reported consequences of β-lactam-induced dysbiosis in children, with aminopenicillins and oral cephalosporins carrying particularly high risk [89,95].

##### Clostridium Difficile-Associated Disease (CDAD)

Although severe CDAD is less common in children than in adults, recent antibiotic exposure—particularly to cephalosporins—consistently appears among the strongest precipitating factors for pediatric CDI in hospitalized patients [90,96,97]. Broad-spectrum antibiotics, including β-lactam agents, have been shown to reduce anaerobic Firmicutes such as members of the *Lachnospiraceae* family, taxa that contribute to secondary bile acid metabolism and colonization resistance. Experimental studies demonstrate that loss of these commensals impairs bile acid–mediated colonization resistance and facilitates *C. difficile* expansion through both metabolic disruption and reduced competitive exclusion [93,98,99]. Although antibiotic exposure—especially cephalosporins—is a well-established risk factor for toxigenic *C. difficile* colonization in hospitalized children, no prospective multicenter studies have identified β-lactams specifically as the strongest independent predictor [90,97,100].

##### Increased Susceptibility to Secondary Infections

Short-term antibiotic-associated dysbiosis may transiently reduce colonization resistance, a phenomenon observed in neonatal intensive care cohorts. In preterm infants exposed to empiric ampicillin–cefotaxime therapy, reductions in *Bifidobacterium* and other obligate anaerobes have been documented, alongside increased abundance of Enterobacteriaceae—organisms frequently implicated in nosocomial colonization [101,102,103,104]. Such pathogen-dominated colonization patterns have been associated with an increased risk of late-onset bloodstream infection in preterm infants, particularly in neonatal intensive care settings. Nevertheless, β-lactam–based regimens (typically a penicillin or aminopenicillin combined with an aminoglycoside) remain the recommended empirical first-line therapy for suspected neonatal sepsis [105,106,107,108].

#### 2.4.2. Long-Term Effects

##### Increased Risk of Atopic Diseases (Eczema, Food Allergy, Allergic Rhinitis)

Early-life antibiotic exposure has been consistently associated with increased risk of childhood allergic and atopic diseases in large population cohorts. These associations are thought to be mediated in part by antibiotic-induced alterations of the developing gut microbiome, although causal pathways remain unproven [109,110,111]. Several pediatric cohort studies have reported associations between antibiotic exposure during the first year of life and a higher risk of allergic outcomes in early childhood, although specific effects by antibiotic class remain difficult to isolate [112,113,114,115]. Early-life antibiotic exposure can delay the establishment of beneficial taxa such as *Bifidobacterium* and other anaerobic fermenters, which contribute to the development of tolerogenic immune pathways. Disruption of SCFA-producing microbial communities during early life may alter microbiota-driven immune signaling involved in regulatory T-cell maturation, potentially biasing immune development toward allergy-prone responses [111,116]. Although observational studies cannot establish causality, the association is biologically plausible, as early-life antibiotic exposure has been shown to disrupt microbial communities involved in regulatory immune development [104,116].

##### Asthma and Recurrent Wheeze

Multiple pediatric cohort and population-based studies have reported associations between early-life antibiotic exposure and an increased risk of asthma or recurrent wheeze in later childhood [112,117]. Early-life alterations in gut microbial composition have been proposed as a potential contributor to immune maturation, including pathways linking the gut and lungs; however, current evidence is largely associative and does not establish a causal mechanism [6,111,118]. During the first months of life, reduced abundance of specific gut bacterial taxa has been associated with an increased risk of later asthma. In parallel, altered microbial metabolites in infancy and enhanced airway inflammatory responses have been demonstrated in experimental models [111].

##### Bronchopulmonary Dysplasia (BPD)

BPD, a chronic respiratory condition predominantly affecting very preterm infants, has been associated with early and prolonged exposure to broad-spectrum antibiotics in the neonatal period. Cohort data indicate that infants exposed to antibiotics for 5–7 days within the first week of life display increased adjusted odds of moderate to severe BPD or mortality compared with no antibiotic exposure. These findings suggest that antibiotic-induced perturbations of early microbial colonization may contribute, at least in part, to persistent pulmonary inflammation and dysregulated immune responses. Emerging evidence supports the role of the gut–lung axis, whereby disruptions in intestinal microbiota influence lung development and inflammatory pathways relevant to BPD pathogenesis [119,120].

##### Obesity and Metabolic Dysregulation

Potential long-term metabolic effects are among the most actively debated consequences of early-life dysbiosis induced by commonly used antibiotics, including β-lactams [3,121,122,123]. Some pediatric cohorts have reported higher body mass index (BMI) or increased risk of overweight among children exposed during infancy to commonly prescribed β-lactam antibiotics, including amoxicillin or cephalosporins [122,124,125]. Antibiotic exposure, including commonly used β-lactam agents, has been shown to reduce gut bacterial taxa involved in SCFAs production and to alter microbial pathways related to bile acid metabolism. Independently, SCFAs and bile acids are recognized regulators of host energy metabolism and appetite signaling [94,126,127]. Early-life dysbiosis characterized by expansion of Gram-negative taxa, including *Enterobacteriaceae*, and reduced abundance of *Bifidobacterium* has been associated with increased intestinal permeability and metabolic endotoxemia—processes implicated in metabolic dysfunction. Observational studies further suggest that these associations may be more pronounced with repeated antibiotic exposure and with courses administered during early infancy [122,125].

##### Long-Term Colonization with Antibiotic-Resistant Bacteria

Repeated exposure to β-lactam antibiotics in children has been associated with prolonged intestinal carriage of antibiotic-resistant *Enterobacteriaceae*. Intestinal colonization with ESBL-producing *Escherichia coli* and *Klebsiella* species has been shown to persist for several months after therapy [128,129,130,131]. Prolonged intestinal colonization with antibiotic-resistant *Enterobacteriaceae* has been associated with an increased risk of subsequent difficult-to-treat infections. In addition, resistant strains frequently spread within households, contributing to the persistence and dissemination of resistance [131,132,133,134].

##### Functional Gastrointestinal Disorders (FGIDs)

There is increasing interest in whether early-life antibiotic-associated dysbiosis may predispose to functional gastrointestinal disorders later in childhood; however, current evidence remains limited and largely observational [82,135]. Experimental studies have shown that antibiotic-induced gut dysbiosis can alter microbial metabolite production, mucosal signaling pathways, and visceral pain processing, supporting their consideration as plausible mediators of later functional gastrointestinal symptoms [136,137,138,139]. While pediatric microbiome data in IBS remain sparse and inconsistent, some cohorts report reduced *Bifidobacterium* and enrichment of *Gammaproteobacteria/Enterobacteriaceae*, patterns that overlap with dysbiosis signatures described after early-life antibiotic exposure [140,141,142,143].

##### Inflammatory Bowel Disease (IBD)

IBD encompasses chronic immune-mediated disorders such as Crohn’s disease and ulcerative colitis, characterized by marked gut microbiome dysbiosis. Epidemiological evidence has linked early-life antibiotic exposure to an elevated risk of pediatric IBD, with antibiotics administered before 2 years of age associated with approximately a 30% increase in adjusted odds of developing IBD in childhood. Disruptions of microbial diversity and composition induced by antibiotics during critical windows of immune maturation may promote pro-inflammatory states and impair mucosal barrier integrity, thereby contributing to disease onset [144,145].

##### Neurodevelopmental and Behavioral Outcomes (Emerging Evidence)

Emerging pediatric data link early-life antibiotic exposure with modest differences in neurodevelopmental outcomes, and experimental and clinical studies on the microbiota–gut–brain axis support the hypothesis that antibiotic-associated dysbiosis could influence brain development. However, class-specific effects of β-lactams and causal mechanisms remain unproven [146,147,148,149,150]. Although epidemiological studies have reported associations between early-life antibiotic exposure and neurodevelopmental outcomes, direct pediatric evidence linking class-specific antibiotic-induced dysbiosis, microbial neurotransmitter pathways, and later neurodevelopment remains sparse, and proposed mechanisms are largely derived from experimental models [146,149,150].

##### Increased Risk of Recurrent Infections

Early-life antibiotic exposure has been shown to disrupt microbiota-dependent immune maturation during critical developmental windows. Experimental and epidemiological data suggest that such perturbations may influence host antimicrobial and antiviral immune responses; however, direct evidence linking β-lactam exposure to increased susceptibility to recurrent infections later in childhood remains limited [6,151,152]. Infants who experience frequent upper respiratory infections are more likely to receive antibiotics early in life, complicating the interpretation of later infection patterns due to confounding by indication [151,153,154]. Experimental and immunological studies indicate that gut dysbiosis can reduce mucosal IgA responses and impair epithelial barrier function, potentially increasing host exposure to microbial antigens and inflammatory signaling [155,156].

### 2.5. Strategies to Mitigate Dysbiosis

#### 2.5.1. Why We Need Antibiotic Stewardship (AS)

Antibiotic stewardship refers to coordinated programs and interventions within health-care settings designed to optimize antibiotic use by ensuring selection of the appropriate agent, dose, route, and duration for each infection, thereby maximizing clinical outcomes while minimizing unnecessary exposure. The primary aims of AS are to improve patient outcomes, reduce antibiotic overuse and misuse, limit the emergence and spread of antimicrobial resistance, and reduce adverse events and costs associated with antimicrobial therapy [157,158].

While originally focused on adults, there is growing recognition that stewardship is critical in pediatric care, given children’s frequent exposure to antibiotics, unique pharmacokinetics, and long lifetime ahead—meaning early antibiotic choices potentially influence their long-term microbial ecology. Implementation of pediatric-targeted AS programs often requires special considerations such as pediatric dosing, developmental pharmacokinetics, age-appropriate diagnostics, and the involvement of multidisciplinary teams (physicians, pharmacists, microbiologists) [159].

AS has become an essential pillar of modern medical practice, not only because of the accelerating threat of antimicrobial resistance, but also due to the growing recognition that broad-spectrum antibiotics fundamentally alter the human microbiome in ways that carry long-term health consequences [160,161]. The concept of dysbiosis—microbial imbalance characterized by loss of diversity, disrupted metabolic networks, and overgrowth of opportunistic pathogens—has shifted from an abstract ecological idea to a clinically meaningful endpoint linked to increased infection risk, immune dysregulation, metabolic disturbances, and chronic inflammatory conditions [162,163]. β-lactam antibiotics, while central to the management of bacterial infections, are among the most frequent triggers of antibiotic-associated dysbiosis because of their widespread use, broad activity against commensal taxa, and repeated prescribing in both hospital and outpatient settings [84,164]. Stewardship therefore represents a primary strategy to reduce unnecessary microbial perturbations by promoting rational, targeted, and evidence-based antibiotic use [157,165].

The need for stewardship is reinforced by studies demonstrating that even short antibiotic courses can reduce microbiome diversity for months, and in some cases for more than a year, with effects that accumulate after repeated exposures [40,84]. Children exposed to antibiotics in the first years of life—a critical window for immune and metabolic programming—show increased risks for asthma, obesity, atopic diseases, and gastrointestinal disorders later in childhood, associations believed to be partially mediated through antibiotic-driven dysbiosis [153,166,167].

Stewardship strategies aim to reduce this unintended microbiome injury by ensuring antibiotics are used only when needed, at the right dose, for the right duration, and with the narrowest effective spectrum. Shorter treatment durations—which have been validated for many common infections such as community-acquired pneumonia, urinary tract infections, and skin/soft-tissue infections—also correlate with more rapid microbiome recovery [168]. Stewardship programs emphasize these evidence-based duration modifications as a core intervention, supporting the principle that “shorter is better” whenever clinically appropriate [157,169].

Another reason AS is essential for mitigating dysbiosis lies in the bidirectional relationship between microbiome health and infection risk. Dysbiosis itself predisposes individuals to colonization by multidrug-resistant organisms (MDROs), including ESBL-producing *Enterobacterales* and vancomycin-resistant enterococci [86,99]. In turn, colonization with MDROs increases the likelihood of future infections requiring even more antibiotics, perpetuating a vicious cycle of microbial damage [170].

By reducing unnecessary antibiotic exposure, stewardship helps preserve colonization resistance—the microbiota’s innate capacity to limit pathogen overgrowth. Evidence shows that repeated or broad-spectrum antibiotic courses can cumulatively disrupt microbial resilience, and such ecological pressure may particularly impair recovery in infants [99,157,164].

In summary, AS represents one of the most effective and immediately actionable strategies to mitigate antibiotic-induced dysbiosis. By limiting unnecessary exposures, optimizing spectrum and duration, and promoting diagnostic accuracy, stewardship programs not only preserve antimicrobial efficacy but are also expected to reduce collateral ecological disruption of the gut microbiome. As understanding of microbiome–host interactions expands, AS is likely to play an increasingly important role in safeguarding both short- and long-term health across the lifespan [151,157,158].

In children, whose gut microbiome undergoes rapid development during infancy and early childhood, unnecessary or broad-spectrum antibiotic exposure can disrupt microbial colonization, diversity, and stability, with potential downstream consequences for immune maturation, metabolic programming, and long-term gut health [2,6,171]. AS can mitigate these risks by ensuring antibiotics are prescribed only when truly indicated, thereby reducing unnecessary perturbations to the developing microbiota through decreased antibiotic exposure. For instance, implementation of AS programs in pediatric clinical settings has been shown to significantly improve the appropriateness of antibiotic choice, dose, and duration for common infections, while reducing excessive use of broad-spectrum agents [157,172,173].

Moreover, in inpatient pediatric settings, AS programs have successfully decreased reliance on broad-spectrum antibiotics in favor of narrower-spectrum or more targeted therapy, without increasing adverse outcomes—a shift that is expected to reduce collateral disruption of commensal gut microbiota [40,174,175]. By limiting total antibiotic exposure—through narrower-spectrum selection, reduced cumulative duration, and avoidance of redundant prescribing—stewardship programs are expected to preserve gut microbial diversity, limit overgrowth of opportunistic taxa such as *Enterobacteriaceae*, and help maintain colonization resistance, a critical ecological barrier against pathogenic bacteria [99,151,164].

Importantly, AS extends beyond resistance containment to safeguarding the ecological integrity of the developing pediatric gut microbiome, which may influence short-term adverse effects (such as AAD) as well as longer-term health trajectories, including immune development, allergy risk, and metabolic health [6,89,157]. Given documented and often persistent alterations in gut microbiome composition observed in children exposed to repeated or long-term antibiotic regimens (e.g., enrichment of *Enterobacteriaceae* and reduction of beneficial *Bifidobacteriaceae*), AS emerges as an essential strategy to balance infection control with microbiome preservation in pediatric practice [82].

#### 2.5.2. Probiotics, Prebiotics, Synbiotics

Antibiotic exposure, particularly courses of β-lactam antibiotics commonly prescribed in pediatrics, disrupts the developing gut ecosystem by reducing microbial diversity, depleting key commensals, and favoring overgrowth of *Proteobacteria* and resistant strains—effects that may persist beyond treatment and motivate active strategies to mitigate dysbiosis in children [84,130,164].

Probiotics—defined as live microorganisms that, when administered in adequate amounts, confer a health benefit on the host—are the most-studied adjunctive approach for preventing antibiotic-associated adverse events such as AAD (AAD) and for aiming to blunt microbiome perturbation during and after antibiotic courses [176]. Multiple systematic reviews and meta-analyses focused on pediatric populations report that specific probiotic strains or combinations (not all products) reduce the risk of AAD with number-needed-to-treat estimates commonly in the single digits, while also lowering mean duration of diarrhoea in those affected [177]. Randomized clinical trials using contemporary molecular microbiome methods demonstrate heterogeneous effects of probiotics on taxonomic diversity and composition. While some studies report small, transient increases in the abundance of supplemented taxa during administration, others show limited or no durable effects on global diversity indices at one month. These findings indicate that observed clinical benefits of probiotics do not necessarily correspond to large compositional shifts detectable by standard taxonomic profiling approaches, including 16S rRNA sequencing [178,179]. Mechanistically, probiotics may act through several, non-exclusive pathways: competitive exclusion of opportunistic taxa, production of antimicrobial metabolites (e.g., lactic acid, bacteriocins), enhancement of mucosal barrier function, modulation of local and systemic immune responses, and even reduction of the gut resistome (antibiotic-resistance gene abundance) in some settings [180,181,182].

The protective potential of probiotics appears to be strain- and dose-dependent. High-quality evidence supports the efficacy of specific strains, particularly *Saccharomyces boulardii* and selected *Lactobacillus* preparations, while some *Bifidobacterium*-containing formulations have also shown benefit. Moreover, several meta-analyses indicate that higher daily doses (for example ≥5 × 10^9^ CFU) are associated with greater effectiveness in preventing AAD [177,183]. Timing and duration appear to influence probiotic effectiveness in practice. In pediatric trials, probiotic supplementation is typically initiated concomitantly with antibiotic therapy and, in many studies, continued for a variable period thereafter; such regimens have been associated with a reduced incidence of AAD. Safety is a critical consideration in children—while probiotics are generally well tolerated in otherwise healthy pediatric populations, rare serious adverse events have occurred in severely immunocompromised or critically ill children, so product choice and patient selection must be guided by clinical context and local expertise [177].

Prebiotics—non-digestible substrates that selectively stimulate the growth or activity of beneficial microbes—represent a complementary or alternative strategy to directly supplying live microbes [184,185]. Common prebiotic compounds used in pediatrics include human milk oligosaccharides (in infants), galacto-oligosaccharides (GOS), and fructo-oligosaccharides (FOS). These substrates are selected for their preferential stimulation of bifidobacteria and other saccharolytic taxa, and are being investigated for their potential to support microbial and metabolic recovery following antibiotic exposure [185,186]. Clinical studies in children show that prebiotic supplementation can increase bifidobacterial abundance and SCFA concentrations and may reduce markers of intestinal inflammation and gut permeability after disturbances, although high-quality trials specifically testing prebiotics as a mitigation strategy following β-lactam exposure remain limited [187,188].

Synbiotics—combined formulations of probiotics plus prebiotics designed to act synergistically—offer a theoretically attractive approach for antibiotic-induced dysbiosis because the prebiotic component may selectively promote the engraftment and function of the co-administered probiotic strain(s) and the indigenous beneficial taxa [189,190]. Compared with probiotics alone, evidence from randomized and observational studies of synbiotics in pediatric populations remains limited. Available trials suggest that synbiotics can induce functional and metabolic changes in the gastrointestinal system of children, with some studies reporting improvements in selected clinical or biochemical markers of gut health; however, robust data on effects following antibiotic exposure, including prevention of AAD or accelerated microbiota recovery, are still lacking [186,191].

Combining a probiotic with an appropriate prebiotic (a synbiotic) can be considered, particularly when the prebiotic is chosen to selectively foster the co-administered probiotic and the child’s tolerability and risk profile are acceptable [192]. Attention should also be paid to the timing of probiotic administration relative to antibiotic dosing. As a pragmatic precaution, some experts recommend separating bacterial probiotic intake from antibiotics to minimize potential loss of viability, although this consideration is not necessary for *Saccharomyces boulardii*, a yeast that is intrinsically resistant to antibacterial agents [193]. In addition to direct nutritional microbial interventions, broader stewardship measures—minimizing unnecessary β-lactam prescriptions, choosing narrow-spectrum agents when clinically appropriate, optimizing dose and duration, and using topical or local therapies instead of systemic antibiotics when feasible—remain fundamental strategies to reduce the incidence and severity of antibiotic-induced dysbiosis at the population level [157,194].

**Figure 3:**
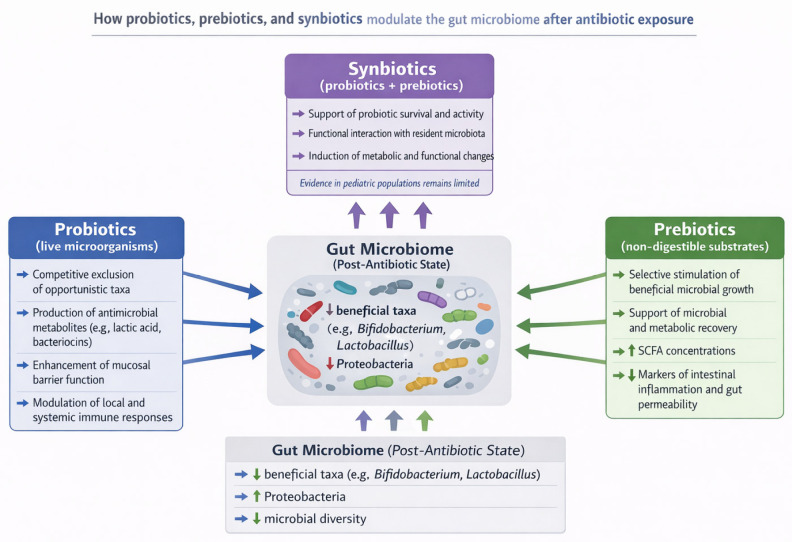
Modulation of the post-antibiotic gut microbiome by probiotics, prebiotics, and synbiotics. Schematic overview of the mechanisms through which probiotics, prebiotics, and synbiotics modulate the gut microbiome following antibiotic exposure. Antibiotic-induced dysbiosis is characterized by a reduction in beneficial taxa (e.g., *Bifidobacterium*, *Lactobacillus*), decreased microbial diversity, and expansion of Proteobacteria. Probiotics primarily exert strain-specific effects via competitive exclusion, antimicrobial metabolite production, and immune modulation, while prebiotics selectively stimulate beneficial commensals, increase short-chain fatty acid concentrations, and support microbial and metabolic recovery. Synbiotics combine both approaches, although evidence in pediatric populations remains limited.

For researchers and clinicians designing mitigation protocols, several practical recommendations emerge from the literature: (i) define clinical endpoints (e.g., AAD incidence, duration, infection recurrence) and microbiome endpoints (diversity metrics, taxa of interest, resistome) a priori; (ii) select probiotic strains and prebiotic substrates with mechanistic plausibility and prior pediatric safety data; (iii) standardize timing (start with antibiotic initiation), dose (use evidence-based CFU thresholds), and duration (continue several weeks post-antibiotic when aiming to reduce AAD); and (iv) incorporate longer follow-up (≥1–3 months) and functional assays (metabolomics, ARG profiling) to capture resilience and recovery rather than only immediate taxonomic shifts [87,177,192]. Finally, when counseling families, emphasize that while microbial adjuncts can reduce the risk of AAD and may help blunt some antibiotic-induced perturbations, they are not a substitute for AS, and product choice should be individualized, evidence-informed, and made in coordination with the child’s primary clinician (Figure 3) [157,195].

### 2.6. Diet-Based Interventions to Support Microbiome Recovery

Dietary strategies are likely to play an important role in supporting microbiome resilience and recovery following antibiotic exposure in childhood, as diet is one of the most potent modulators of microbial composition and metabolic activity during microbiome development across pediatric age groups [196]. A higher intake of dietary fiber is consistently associated with increased gut microbial diversity and enrichment of beneficial taxa such as *Bifidobacterium* and short-chain–fatty-acid–producing *Firmicutes*, taxa that are commonly reduced in states of antibiotic-associated dysbiosis [196,197,198]. Fiber-rich foods, including fruits, vegetables, legumes, and whole grains, provide fermentable substrates that promote the production of SCFAs, key metabolites involved in epithelial repair, immune regulation, and restoration of gut homeostasis [199,200]. Although dietary fiber intake is known to increase the abundance of SCFA-producing taxa and SCFA levels in children, direct evidence linking fiber consumption to accelerated SCFA recovery following antibiotic exposure remains limited. Nevertheless, mechanistic insights and observational patterns—largely derived from developmental and adult recovery studies—suggest a potential supportive role [84,196,197]. In addition to fiber, foods naturally containing prebiotic compounds—such as bananas, asparagus, onions, leeks, and wheat-based products—provide fructo- and galacto-oligosaccharides, which are known to selectively stimulate beneficial taxa such as *Bifidobacterium* [192,201,202].

While supplemental prebiotics—particularly FOS and GOS—have been more extensively studied, foods naturally rich in prebiotic compounds (e.g., bananas, onions, leeks, wheat-based products) also contain substrates that selectively stimulate *Bifidobacterium*. This effect may be particularly relevant during early childhood, a period in which the gut microbiome is still developing and remains highly responsive to dietary inputs [201,202,203]. Fermented foods such as yogurt and kefir contain live microorganisms, and randomized trials in adults show that increased consumption of fermented foods can transiently increase gut microbial diversity. Certain fermented dairy products can also inhibit pathogen growth, suggesting potential contributions to host colonization resistance; however, direct evidence for these effects in children or in the post-antibiotic setting remains limited [204,205].

Dietary patterns modulate intestinal inflammatory responses, and experimental studies—primarily in animal models—demonstrate that fiber-deprived or high-fat diets increase intestinal permeability. Such changes may predispose to worsened barrier dysfunction following antibiotic-associated microbiota disruption; however, direct clinical evidence in children remains limited [206,207,208].

Diet-based interventions should be individualized according to developmental stage, dietary limitations, and gastrointestinal tolerability. Whole-food sources of fiber and naturally occurring prebiotics are generally better tolerated in younger children, whereas adolescents—whose microbiome and digestive capacity more closely resemble those of adults—may tolerate larger amounts of fermentable fibers with fewer gastrointestinal symptoms [202,209]. Although dietary modification alone cannot fully reverse antibiotic-induced dysbiosis, diet represents a low-risk and biologically plausible means of supporting microbiome recovery. Available evidence indicates that dietary approaches interact with and complement probiotic, prebiotic, and antibiotic-stewardship strategies, together contributing to a more favorable ecological rebound following antibiotic exposure [84,200,210].

## 3. Controversies

### 3.1. Individual Variability & the “Healthy Microbiome” Concept

It is important to note that the concept of ‘dysbiosis’ remains debated. Most human microbiome studies—including those assessing antibiotic effects—are descriptive and lack a validated definition of a ‘healthy’ microbiome [211]. This limits our ability to definitively state that observed shifts after antibiotic exposure are pathological rather than benign alternative microbial equilibria. Many shifts traditionally labeled as dysbiosis may instead reflect underlying host-driven ecological changes—including altered oxygenation, inflammation, or increased availability of host-derived electron acceptors—rather than pathogenic microbes themselves [212]. Furthermore, a number of documented links between altered microbiota and illness are still correlational, complicated not only by host genetics but also by exposure to the environment, food, and other factors. According to this viewpoint, dysbiosis is often a result of physiological disruption rather than its primary cause [212,213]. Thus, while antibiotic-associated microbiome alterations raise concern, claims of causality or long-term harm must remain cautious.

### 3.2. Overinterpretation of Animal Studies

Numerous mechanistic insights are derived from germ-free or antibiotic-treated mouse models, even those used in this paper, particularly those that connect dysbiosis to immune tolerance, obesity, or neurodevelopment. It is controversial to translate these findings to humans because of significant physiological and ecological differences [214].

### 3.3. Confounding by Indication

This is an important constraint in perinatal microbiome research. Antibiotics are frequently administered to pregnant patients due to diseases such as urinary tract infections, chorioamnionitis, or preterm rupture of membranes. These disorders have independent effects on neurodevelopment, metabolic risk, birth outcomes, and neonatal immune development. As a result, research may incorrectly associate antibiotic exposure with negative outcomes when the true underlying causes are the mother’s infection or inflammatory state. It is challenging to completely account for the complicating factor, particularly when infection severity, timing, or subclinical inflammation are not sufficiently assessed. Therefore, rather than a direct microbiome-mediated effect of antibiotics, associations between prenatal antibiotic use and outcomes like asthma, obesity, low birth weight, or neurodevelopmental disorders may partially reflect maternal susceptibility, immune dysregulation, or intrauterine inflammatory exposures [215,216].

### 3.4. Over-Sensationalization Issue

Despite the rapid growth of microbiome research, the field remains prone to over-sensationalization. Media coverage and some scientific publications often frame microbiome alterations as direct causes of disease, overlooking the complexity of host–microbe interactions.

Due to limited sample sizes, diverse populations, and variations in sequencing or analytical methods, many observed relationships are correlational rather than causal. As a result, research findings are often not replicable. Despite evidence that great diversity is not always advantageous and may even be harmful in some situations, such as the vaginal microbiota, metrics like microbial diversity are frequently interpreted simplistically as markers of health or dysbiosis. Additionally, there are inconsistent findings regarding the functional effects of changes in the microbiome, and the influence of variables such as environment, genetics, and diet is often overlooked.

## 4. Conclusions

Perinatal antibiotic exposure can significantly impact both the maternal and neonatal microbiomes, with potential effects on immune development, metabolic programming, and neurodevelopment in offspring. Although evidence has linked this exposure to increased risks of asthma, obesity, and altered neurodevelopmental trajectories, it is important to note that many of these findings are correlational rather than causal. Therefore, confounding factors such as maternal infections, genetics, and environmental influences must be carefully considered. The field of microbiome research is promising, yet it is also susceptible to exaggerated claims and inconsistent results, making cautious interpretation necessary—especially regarding simplistic measures like microbial diversity. To clarify causality and inform safe clinical interventions, future studies should be mechanistic, longitudinal, and context-aware, integrating host, microbial, and environmental factors. Given the widespread use of β-lactam antibiotics in pediatrics and obstetrics, implementing stewardship strategies that emphasize appropriate indication, narrow spectrum when feasible, and minimal effective duration may help mitigate unintended microbiome alterations while maintaining clinical efficacy. Ultimately, a nuanced understanding of the maternal-fetal microbiome axis will be essential for optimizing maternal and child health, avoiding unnecessary alarm, and preventing premature therapeutic interventions.

## Figures and Tables

**Table 1:** Reported changes in major gut bacterial taxa across early-life conditions and β-lactam exposure. This table summarizes reported patterns of relative abundance changes in major gut bacterial taxa across key early-life conditions and following exposure to β-lactam antibiotics. The table synthesizes findings described in the text and referenced literature, highlighting taxa consistently reported as increased or decreased under each condition. The information is intended as a qualitative overview rather than a causal or quantitative comparison.

Condition/Exposure	Increased Taxa (Reported)	Decreased Taxa (Reported)	References
Vaginal delivery	*Lactobacillus*, *Prevotella*, *Sneathia*	-	[12]
Caesarean section	*Staphylococcus*, *Corynebacterium*, *Propionibacterium*	*Lactobacillus*, *Bifidobacterium*	[12]
Breastfeeding	*Bifidobacterium*, *Lactobacillus*	*-*	[16,18]
Formula feeding	*Escherichia coli*, *Clostridioides difficile*, *Bacteroides*, *Prevotella*	*Bifidobacterium*	[3,18]
Intrapartum antibiotic prophylaxis (IAP)	*Enterobacteriaceae*, *Enterococcaceae*	*Lactobacillus*	[26]
Early postnatal antibiotic exposure	*Enterobacteriaceae*, *Enterococcaceae*	*Bifidobacterium*, *Bacteroides*	[25,27]
Aminopenicillins (±β-lactamase inhibitors)	*Enterobacteriaceae*	*Bifidobacterium*, *Lactobacillus*	[28,29]
Broad-spectrum β-lactams (e.g., 3rd gen cephalosporins)	*Enterococcaceae*	*Bifidobacterium*, *Lactobacillus*	[29,30]

**Table 2:** Spectrum of β-Lactam Antibiotics and Their Effects on Gut Microbial Communities.

Sub-Class	Representative Agents	Main Antimicrobial Targets	Typical Pediatric Use	Microbiome Effects	Notes/References
Narrow-spectrum Penicillins	Benzylpenicillin, Phenoxymethylpenicillin	*Streptococcus*, *Staphylococcus* (non–β-lactamase producers)	Streptococcal pharyngitis, mild skin infections	Minimal impact; limited reduction in Gram-positive commensals; microbiome recovery within months	[28]
Aminopenicillins ± β-lactamase inhibitors	Amoxicillin, Ampicillin, Amoxicillin–clavulanate	Broader Gram-positive & some Gram-negative (*H. influenzae*, *E. coli*)	Otitis media, sinusitis, respiratory & urinary infections	↓ *Bifidobacterium*, ↓ *Lactobacillus*, ↑ *Proteobacteria*, ↓ diversity	[28,29]
1st-Generation Cephalosporins	Cefalexin, Cefazolin	Gram-positive cocci, limited Gram-negative (*E. coli*, *Klebsiella*)	Skin/soft tissue infections	Moderate reduction of Gram-positive commensals. ↑ *Enterobacteriaceae*; transient dysbiosis	[30]
2nd-Generation Cephalosporins	Cefuroxime, Cefaclor	Gram-positive + expanded Gram-negative (*H. influenzae*, *Moraxella*)	Respiratory infections	Loss in taxonomic variety could be observed; ↑ *Enterococcaceae* and *Enterobacteriaceae*	[43,44]
3rd-Generation Cephalosporins	Ceftriaxone, Cefotaxime, Cefixime	Broad Gram-negative incl. *Enterobacteriaceae*; reduced Gram-positive	Severe respiratory or meningeal infections	↓ *Bifidobacterium*, ↓ *Lactobacillus *↑ *Enterococcus*, ↑ *Candida*	[45,46]
Carbapenems	Meropenem, Imipenem, Ertapenem	Very broad: Gram-positive, Gram-negative, anaerobes, ESBL strains	Severe or resistant infections	Severe depletion of *Bifidobacterium;* Loss in taxonomic variety; ↑ *Enterobacteriaceae*;	[47]
Monobactams	Aztreonam	Aerobic Gram-negative bacteria	Gram-negative infections (esp. β-lactam allergy cases)	Minimal dysbiosis; limited data	[48]

## Data Availability

No new data were created or analyzed in this study. Data sharing is not applicable to this article.

## Abbreviations

AAD	Antibiotic-associated diarrhea
ARG	Antibiotic resistance gene
AS	Antibiotic stewardship
ASD	Autism spectrum disorder
β-lactams	Beta-Lactams
BMI	Body mass index
BPD	Bronchopulmonary dysplasia
CDAD	Clostridium difficile–associated disease
CDI	Clostridium difficile infection
CELSPAC: TNG	Central European Longitudinal Studies of Parents and Children: The Next Generation
CFU	Colony-forming units
ESBL	Extended-spectrum β-lactamase
FGIDs	Functional gastrointestinal disorders
FOS	Fructo-oligosaccharides
GBS	Group B Streptococcus
GOS	Galacto-oligosaccharides
HMOs	Human milk oligosaccharides
HPA	Hypothalamic–pituitary–adrenal
IAP	Intrapartum antibiotic prophylaxis
IBD	Inflammatory bowel disease
IBS	Irritable bowel syndrome
MDROs	Multidrug-resistant organisms
MRSA	Methicillin-resistant *Staphylococcus aureus*
NICU	Neonatal intensive care unit
SCFAs	Short-chain fatty acids
